# Case-control study of *IL13 *polymorphisms, smoking, and rhinoconjunctivitis in Japanese women: the Kyushu Okinawa Maternal and Child Health Study

**DOI:** 10.1186/1471-2350-12-143

**Published:** 2011-10-25

**Authors:** Yoshihiro Miyake, Keiko Tanaka, Masashi Arakawa

**Affiliations:** 1 Department of Preventive Medicine and Public Health, Faculty of Medicine, Fukuoka University, Fukuoka, Japan; 2 Field Science for Health and Recreation, Faculty of Tourism Sciences and Industrial Management, University of the Ryukyus, Okinawa, Japan

## Abstract

**Background:**

Six previous studies have examined the relationships between single nucleotide polymorphisms (SNPs) in the *IL13 *gene and allergic rhinitis, but the results have been inconsistent. However, a recent meta-analysis using data from these 6 studies has shown that the A allele of *IL13 *SNP rs20541 was associated with an increased risk of allergic rhinitis, whereas no such relationship existed between *IL13 *SNP rs1800925 and allergic rhinitis. We investigated the associations between *IL13 *SNPs rs1800925 and rs20541 and the risk of rhinoconjunctivitis in Japanese women.

**Methods:**

Included were 393 cases who met the criteria of the International Study of Asthma and Allergies in Childhood (ISAAC) for rhinoconjunctivitis. Control subjects were 767 women without rhinoconjunctivitis according to the ISAAC criteria, who had also not been diagnosed with allergic rhinitis by a doctor. Adjustment was made for age, region of residence, presence of older siblings, smoking, family history of allergic rhinitis, and education.

**Results:**

Compared with the GG genotype of *IL13 *SNP rs20541, the AA genotype, occurring in 7.1% of control subjects, was significantly positively related to the risk of rhinoconjunctivitis: the adjusted odds ratio was 1.65 (95% confidence interval: 1.05 - 2.60). SNP rs1800925 was not associated with rhinoconjunctivitis. The haplotype comprising the rs1800925 C allele and the rs20541 A allele was significantly positively related to rhinoconjunctivitis. The multiplicative interactions between the two SNPs under study and smoking on the risk of rhinoconjunctivitis were not statistically significant. Based on the recessive model, however, the additive interaction between SNP rs1800925, but not rs20541, and smoking was significant.

**Conclusions:**

This study suggests that the minor genotype of *IL13 *SNP rs20541 and the CA haplotype are significantly positively associated with the risk of rhinoconjunctivitis. In addition, a new pattern of biological interaction that affects the risk of rhinoconjunctivitis is described between SNP rs1800925 and smoking.

## Background

Allergic rhinitis is the most common allergic condition in Japan. One study of 1540 Japanese aged 20 to 49 years showed that the prevalence of allergic rhinitis was 44.2% and that 89.6% of the subjects with allergic rhinitis were sensitized to Japanese cedar pollen [1]. In a nationwide survey in Japan, the age-adjusted prevalence of Japanese cedar pollinosis was 19.4%; the prevalence was higher in urban areas than in rural areas [2]. The development of allergic rhinitis entails an interplay between genetic predisposition and a range of environmental exposures.

Interleukin (IL)-13 is a critical mediator in the pathogenesis of allergic inflammation [3]. One study has revealed increased expression of the IL-13 gene in the epithelial compartment of the nasal mucosa of patients with perennial allergic rhinitis, and no such increase in the nasal epithelial compartment of control subjects [4].

The *IL13 *gene is located in the chromosome 5q31-33 region. A recent meta-analysis incorporating 6 independent studies of children and adults [5-10] reported that the A allele of *IL13 *single nucleotide polymorphism (SNP) rs20541 (Arg110/130Glu) in the coding region of exon 4 was associated with an increased risk of allergic rhinitis whereas *IL13 *SNP rs1800925 (-1024C/T) in the promoter region was not related to the risk of allergic rhinitis [11]. It should be noted, however, that the 6 studies included in that meta-analysis provided inconsistent results [5-10].

Here, we conducted a case-control study to investigate the relationships between *IL13 *SNPs rs1800925 and rs20541 and the risk of rhinoconjunctivitis in Japanese women using data from the Kyushu Okinawa Maternal and Child Health Study (KOMCHS). In addition, we carried out haplotype analyses and examined the possibility of an interaction between the SNPs and smoking. IL-13 production has been shown to be significantly greater in smokers than in non-smokers [12]. A significant interaction was observed between SNP rs1800925 and smoking, with allergy as its outcome, in UK adults [5].

## Methods

### Study Population

The KOMCHS is an ongoing prospective prebirth cohort study that investigates preventive and risk factors for maternal and child health problems such as allergic disorders. From April 2007 to March 2008, the KOMCHS requested that 131 obstetric hospitals in Fukuoka Prefecture, the largest prefecture on Kyushu Island in southern Japan, with a total population of approximately 5.04 million, provide as many pregnant women as possible with a set of leaflets explaining the KOMCHS, an application to participate in the study, and a self-addressed stamped envelope in which to return the application. From May 2007 to March 2008, the KOMCHS also requested that 40 obstetric hospitals in Okinawa Prefecture, one of the southernmost islands of Japan, with a total population of almost 1.37 million, provide as many pregnant women as possible with a comparable set of documents. Later, to increase the sample size, pregnant women living in 6 prefectures on Kyushu Island other than Fukuoka Prefecture, with a total population of approximately 8.22 million, were provided with comparable documents at 252 obstetric hospitals between August 2007 and March 2008. Pregnant women who intended to participate in the KOMCHS returned the application form to the data management center. By the end of the baseline study, a total of 1757 pregnant women between the 5th and 39th week of pregnancy had given their fully informed consent in writing to participate in the KOMCHS and had completed the baseline survey. Around four months after delivery, 1492 women gave informed consent to genotyping. The ethics committee of the Faculty of Medicine, Fukuoka University, approved the KOMCHS.

### Selection of Cases and Control Subjects

In the baseline survey, each participant filled out a self-administered questionnaire and mailed the completed questionnaire to the data management center. Research technicians completed missing or illogical data by telephone interview.

The questionnaire included questions on rhinoconjunctivitis based on the International Study of Asthma and Allergies in Childhood (ISAAC) [13]. The presence of rhinoconjunctivitis was defined as a positive response to the following 2 questions: 'In the last 12 months, have you had a problem with sneezing or a runny or blocked nose when you did not have a cold or flu?' and 'In the last 12 months, has this nose problem been accompanied by itchy-watery eyes?' The questionnaire also elicited information on age, region of residence, presence of older siblings, smoking habits, family history of allergic rhinitis, and education. A history of smoking was defined as having smoked at least once per day for at least one year. A family history of allergic rhinitis (including Japanese cedar pollinosis) was considered to be present if one or more parents or siblings of the study subjects had been diagnosed with allergic rhinitis by a physician.

By this definition, there were 393 cases of rhinoconjunctivitis among the 1492 participants whose DNA samples were available. Among the remaining participants, who would serve as control subjects, 331 women were excluded who were not considered to have rhinoconjunctivitis as defined by the ISAAC criteria but who had answered 'yes' to the question: 'Have you ever been diagnosed by a physician as having allergic rhinitis?'. Incomplete data on smoking caused the exclusion of 1 additional woman. In the end, 767 participants were identified as control subjects for our analysis.

We also performed a sensitivity analysis restricted to the 293 cases with doctor-diagnosed allergic rhinitis out of the 393 cases considered to have rhinoconjunctivitis based on the ISAAC criteria.

### DNA Extraction and Genotyping

Research technicians or subjects themselves collected buccal specimens with BuccalAmp swabs (Epicenter BioTechnologies, Madison, WI, USA). Genomic DNA was extracted using a QIAmp DNA mini kit (Qiagen, Inc., Valencia, CA, USA). Two SNPs from the *IL13 *gene were used in this study: rs1800925 in the 5' promoter region and rs20541 in exon 4. Genotyping of the two *IL13 *polymorphisms was performed using pre-development TaqMan SNP Genotyping Assays (Applied Biosystems, Foster City, CA, USA). Information on SNP rs20541 in one control subject was missing because genotype identification was impossible.

### Statistical Analysis

Deviation from the Hardy-Weinberg equilibrium among control subjects was evaluated by the chi-square test. Linkage disequilibrium was investigated using Haploview software version 4.1 [14]. Estimations of crude odds ratios (ORs) and 95% confidence intervals (CIs) for rhinoconjunctivitis in relation to the SNPs under study were made by means of logistic regression analysis under additive (Model 1) and dominant (Model 2) models. Multiple logistic regression analysis was used to adjust for age, region of residence, presence of older siblings, smoking, family history of allergic rhinitis, and education. The presence of older siblings was significantly associated with a lower prevalence of rhinoconjunctivitis in this population [15]. Haplotypes and their frequencies were inferred with the expectation maximization (EM) algorithm. For differences in haplotype frequency between the case and control groups, crude ORs and 95% CIs were estimated based on the frequency of each haplotype relative to all other haplotypes combined. The multiplicative interaction was estimated by introducing a multiplicative term into a multiple logistic regression model. Three measures were used to test the additive interaction [16]: 1) relative excess risk due to interaction (RERI), 2) attributable proportion due to interaction (AP), and 3) synergy index (S). RERI is the excess risk due to an interaction relative to the risk without exposure. AP refers to the attributable proportion of disease that is due to an interaction among individuals with both exposures. S is the excess risk from both exposures when there is an additive interaction, relative to the risk from both exposures without an interaction. If any of the null values (0 in RERI and AP or 1 in S) falls outside the 95% CI of its respective measurement, then the additive interaction is considered statistically significant. Details of the method for the calculation of the additive interaction have been described by Andersson et al. [16]. Excluding the calculation of linkage disequilibrium, all computations were performed using STATA/SE software version 11.0 (StataCorp, College Station, TX, USA).

## Results

Compared with control subjects, women with rhinoconjunctivitis were more likely to live in prefectures other than Fukuoka Prefecture on Kyushu Island and to have a family history of allergic rhinitis, and were less likely to live in Okinawa Prefecture and to have one or more older siblings (Table 1). There were no differences between cases and control subjects with regard to age, smoking, and education.

**Table 1:** Characteristics of the study population

	*n *(%) or mean (SD)		
	
Variable	Cases (*N *= 393)	Controls (*N *= 767)	*P *value*
Age (years)	31.4 (4.2)	31.3 (4.3)	0.73
Region of residence (%)			0.01
Fukuoka Prefecture	221 (56.2)	438 (57.1)	
Other than Fukuoka Prefecture in Kyushu	148 (37.7)	245 (31.9)	
Okinawa Prefecture	24 (6.1)	84 (11.0)	
Presence of one or more older siblings (%)	181 (46.1)	419 (54.6)	0.006
Having ever smoked (%)	125 (31.8)	224 (29.2)	0.36
Family history of allergic rhinitis (%)	224 (57.0)	248 (32.3)	< 0.0001
Education (% years)			0.66
< 13	93 (23.7)	168 (21.9)	
13 - 14	133 (33.8)	253 (33.0)	
≥ 15	167 (42.5)	346 (45.1)	

* χ^2 ^test or t test.

The distributions of *IL13 *SNPs rs1800925 and rs20541 among control subjects were in Hardy-Weinberg equilibrium (*P *= 0.54 and 0.11, respectively). SNP rs1800925 showed a high linkage disequilibrium value with SNP rs20541 (*D*' = 0.84).

Compared with the CC genotype of SNP rs1800925, neither the CT nor the TT genotype was significantly associated with the risk of rhinoconjunctivitis (Table 2). No significant relationship was observed between the combination of CT and TT genotypes of rs1800925 and rhinoconjunctivitis. On the other hand, the frequency of the AA genotype of SNP rs20541 was 10.9% in cases and 7.1% in control subjects. Using women with the GG genotype as a reference group, those with the AA genotype had a significantly increased risk of rhinoconjunctivitis, whereas the GA genotype was not associated with the risk of rhinoconjunctivitis in crude analysis. After adjustment for age, region of residence, presence of older siblings, smoking, family history of allergic rhinitis, and education, the positive association between the AA genotype and rhinoconjunctivitis remained: the adjusted OR was 1.65 (95% CI: 1.05 - 2.60). Nevertheless, there was no significant relationship between the GA and AA genotypes combined and rhinoconjunctivitis.

**Table 2:** Odds ratios (ORs) and 95% confidence intervals (CIs) for rhinoconjunctivitis according to *IL13 *polymorphisms in Japanese women, KOMCHS, Japan

SNP	Genotype	Cases *n *(%)	Controls *n *(%)	Crude OR (95% CI)	Adjusted OR (95% CI)*
rs1800925		(*N *= 393)	(*N *= 767)		
Model 1	CC	270 (68.7)	516 (67.3)	1.00	1.00
	CT	110 (28.0)	230 (30.0)	0.91 (0.70 - 1.20)	0.94 (0.71 - 1.25)
	TT	13 (3.3)	21 (2.7)	1.18 (0.58 - 2.40)	1.10 (0.53 - 2.28)
Model 2	CC	270 (68.7)	516 (67.3)	1.00	1.00
	CT + TT	123 (31.3)	251 (32.7)	0.94 (0.72 - 1.22)	0.96 (0.73 - 1.26)
rs20541		(*N *= 393)	(*N *= 766)		
Model 1	GG	185 (47.1)	379 (49.5)	1.00	1.00
	GA	165 (42.0)	333 (43.5)	1.02 (0.79 - 1.31)	1.03 (0.79 - 1.34)
	AA	43 (10.9)	54 (7.1)	1.63 (1.05 - 2.53)	1.65 (1.05 - 2.60)
Model 2	GG	185 (47.1)	379 (49.5)	1.00	1.00
	GA + AA	208 (52.9)	387 (50.5)	1.10 (0.86 - 1.40)	1.12 (0.86 - 1.44)

* Adjusted for age, region of residence, presence of older siblings, smoking, family history of allergic rhinitis, and education.

In a sensitivity analysis restricted to cases with doctor-diagnosed allergic rhinitis (n = 293), the results were similar to those observed in the overall analysis (Table 3). The adjusted OR for the AA genotype of SNP rs20541 was 1.66 (95% CI: 1.004 - 2.75).

**Table 3:** Odds ratios (ORs) and 95% confidence intervals (CIs) for doctor-diagnosed allergic rhinitis according to *IL13 *polymorphisms in Japanese women, KOMCHS, Japan

SNP	Genotype	Cases *n *(%)	Controls *n *(%)	Crude OR (95% CI)	Adjusted OR (95% CI)*
rs1800925		(*N *= 293)	(*N *= 767)		
Model 1	CC	201 (68.6)	516 (67.3)	1.00	1.00
	CT	84 (28.7)	230 (30.0)	0.94 (0.70 - 1.26)	0.97 (0.71 - 1.33)
	TT	8 (2.7)	21 (2.7)	0.98 (0.43 - 2.24)	0.89 (0.38 - 2.13)
Model 2	CC	201 (68.6)	516 (67.3)	1.00	1.00
	CT + TT	92 (31.4)	251 (32.7)	0.94 (0.70 - 1.26)	0.96 (0.71 - 1.30)
rs20541		(*N *= 293)	(*N *= 766)		
Model 1	GG	135 (46.1)	379 (49.5)	1.00	1.00
	GA	126 (43.0)	333 (43.5)	1.06 (0.80 - 1.41)	1.08 (0.80 - 1.46)
	AA	32 (10.9)	54 (7.1)	1.66 (1.03 - 2.69)	1.66 (1.004 - 2.75)
Model 2	GG	135 (46.1)	379 (49.5)	1.00	1.00
	GA + AA	158 (53.9)	387 (50.5)	1.15 (0.88 - 1.50)	1.16 (0.88 - 1.55)

* Adjusted for age, region of residence, presence of older siblings, smoking, family history of allergic rhinitis, and education.

The haplotype containing the rs1800925 C allele and the rs20541 A allele was significantly associated with an increased risk of rhinoconjunctivitis, as compared with all other haplotypes combined (Table 4). The other haplotypes were not related to rhinoconjunctivitis.

**Table 4:** Haplotype analysis of rs1800925 and rs20541 in relation to rhinoconjunctivitis in Japanese women, KOMCHS, Japan

Haplotype	Haplotype structure		Frequency *n *(%)		
		
number	rs1800925	rs20541	Cases (2*N *= 786)	Controls (2*N *= 1534)	Crude OR (95% CI)*
1	C	G	516 (65.6)	1068 (69.6)	0.83 (0.69 - 1.01)
2	C	A	134 (17.0)	194 (12.6)	1.42 (1.11 - 1.81)
3	T	G	19 (2.4)	25 (1.6)	1.50 (0.77 - 2.84)
4	T	A	117 (14.9)	247 (16.1)	0.91 (0.71 - 1.16)

* Crude OR for each haplotype is relative to all other haplotypes combined.

A positive relationship between the TT genotype of SNP rs1800925 and rhinoconjunctivitis was detected in women who had a history of smoking, but not in those who had never smoked, although this positive relationship was not statistically significant (Table 5). A positive association between the AA genotype of SNP rs20541 and rhinoconjunctivitis was more pronounced in women who had smoked than in those who had never smoked; nevertheless, the positive associations were not significant regardless of smoking status. There were no significant multiplicative interactions between the SNP rs1800925 or rs20541 and smoking with regard to rhinoconjunctivitis.

**Table 5:** Association between *IL13 *polymorphisms and rhinoconjunctivitis, stratified by smoking status in Japanese women, KOMCHS, Japan

		Smoking status				
			
		No		Yes		
			
SNP	Genotype	No. cases/controls	Adjusted OR (95% CI)*	No. cases/controls	Adjusted OR (95% CI)*	*P *for interaction
rs1800925						
Model 1	CC	185/370	1.00	85/146	1.00	
	CT	75/156	1.08 (0.76 - 1.52)	35/74	0.76 (0.46 - 1.25)	0.30
	TT	8/17	0.75 (0.30 - 1.87)	5/4	2.48 (0.63 - 9.74)	0.17
Model 2	CC	185/370	1.00	85/146	1.00	
	CT + TT	83/173	1.04 (0.74 - 1.45)	40/78	0.84 (0.52 - 1.35)	0.53
rs20541						
Model 1	GG	129/275	1.00	56/104	1.00	
	GA	113/227	1.16 (0.84 - 1.60)	52/106	0.82 (0.50 - 1.34)	0.28
	AA	26/40	1.46 (0.83 - 2.56)	17/14	2.10 (0.94 - 4.67)	0.37
Model 2	GG	129/275	1.00	56/104	1.00	
	GA + AA	139/267	1.20 (0.88 - 1.64)	69/120	0.98 (0.62 - 1.55)	0.52

* Adjusted for age, region of residence, presence of older siblings, family history of allergic rhinitis, and education.

Based on the recessive model, the additive interactions between *IL13 *SNPs and smoking were examined (Table 6). Compared with women with the CC or CT genotype of SNP rs1800925 who had never smoked, those with the TT genotype who had ever smoked had a 2.67-fold increased risk of rhinoconjunctivitis, although the adjusted OR was not significant. The estimated AP, however, was statistically significant (AP = 0.71, 95% CI: 0.25 - 1.18), though RERI and S were not. Compared with women with the GG or GA genotype of SNP rs20541 who had never smoked, those with the AA genotype who had ever smoked showed a significantly increased risk of rhinoconjunctivitis, although the additive interaction was not significant.

**Table 6:** Additive interaction between *IL13 *polymorphisms and smoking

	Smoking status	
	
	No	Yes
	
SNP	Adjusted OR (95% CI)*	Adjusted OR (95% CI)*
rs1800925		
CC + CT	1.00	0.98 (0.74 - 1.31)
TT	0.78 (0.32 - 1.89)	2.67 (0.70 - 10.17)
Measures of additive interaction^†^		
Relative excess risk due to interaction (RERI) = 1.90 (95% CI: -1.71 - 5.52)		
Attributable proportion due to interaction (AP) = 0.71 (95% CI: 0.25 - 1.18)		
Synergy index (S) = -6.97 (95% CI: not calculable)		
rs20541		
GG + GA	1.00	0.96 (0.71 - 1.29)
AA	1.34 (0.78 - 2.29)	2.34 (1.10 - 4.98)
Measures of additive interaction^†^		
Relative excess risk due to interaction (RERI) = 1.05 (95% CI: -0.82 - 2.91)		
Attributable proportion due to interaction (AP) = 0.45 (95% CI: -0.07 - 0.96)		
Synergy index (S) = 4.52 (95% CI: 0.25 - 83.47)		

* Adjusted for age, region of residence, presence of older siblings, family history of allergic rhinitis, and education.

^† ^Statistically significant with the 95% CI of RERI > 0, the 95% CI of AP > 0, or the 95% CI of S > 1, indicating additive interaction.

## Discussion

The current study found that, compared with the GG genotype of *IL13 *SNP rs20541, the AA genotype, occurring in 7.1% of control subjects, was significantly positively related to the risk of rhinoconjunctivitis, whereas the GA genotype and the GA and AA genotypes combined were not associated with rhinoconjunctivitis. In contrast, *IL13 *SNP rs1800925 was not materially related to the risk of rhinoconjunctivitis. Haplotype analyses found a significant positive relationship between the haplotype carrying the rs1800925 C allele and the rs20541 A allele, occurring in 12.6% of control subjects, and the risk of rhinoconjunctivitis. The multiplicative interactions between the two SNPs under study and smoking did not have a statistically significant effect on the risk of rhinoconjunctivitis. Based on the recessive model, however, the additive interaction between SNP rs1800925, but not rs20541, and smoking was significant, as the AP value indicates.

A case-control study of Korean young adults found a significant positive association between the rs20541 A allele and allergic rhinitis, whereas the positive relationship between the AA genotype and allergic rhinitis was of borderline significance [7]. In a case-control study in Japan, on the other hand, SNP rs20541 was not related to the risk of Japanese cedar pollinosis [8]. No significant association was observed between SNP rs20541 and hay fever in a case-control study of German adults [9]. Neither SNP rs1800925 nor rs20541 was associated with hay fever or allergic rhinitis in studies in the UK [5], Spain [6], and China [10]. These inconsistent results may be at least partly explained by differences in the genetic backgrounds of the populations examined, definitions of allergic rhinitis, and statistical power. Yet when the data from these six studies [5-10] were combined in a meta-analysis, they indicated a significant positive association between the rs20541 A allele and allergic rhinitis (overall OR using a fixed-effect model = 1.18 [95% CI: 1.05 - 1.31]), and no association between SNP rs1800925 and allergic rhinitis (overall OR using a fixed-effect model = 1.09 [95% CI: 0.94 - 1.27]) [11]. The current findings are in agreement with the results of this meta-analysis.

A study in Japan showed that schoolchildren with the AA genotype of SNP rs20541 had significantly higher levels of serum IL-13 than those with the GG genotype [17]. Another study in Japan found that the IL-13 Glu110 variant had a lower affinity with IL-13Rα2 than with wild-type IL-13, causing less clearance and enhanced stability in plasma [18]. Vladich et al. have shown that the IL-13 Glu110 variant is significantly more active than wild-type IL-13 in inducing STAT6 phosphorylation and CD23 expression in primary human monocytes and hydrocortisone-dependent IgE switching in human B cells [19]. That paper also reported that a soluble form of IL-13Rα2 neutralized wild-type IL-13 more effectively than it did the IL-13 Glu110 variant [19]. A study in the Netherlands observed that the TT genotype of SNP rs1800925 was significantly associated with reduced inhibition of IL-13 production by anti-CD2, compared with the CT or CC genotype [20]. Cameron et al. have shown that the T allele of SNP rs1800925 increased *IL13 *transcription in primary human and murine CD4^+ ^Th2 lymphocytes through Yin-Yang 1-dependent attenuation of STAT6-mediated promoter repression, and that mitogen-activated peripheral blood mononuclear cells from pregnant women with the TT genotype of SNP rs1800925 secreted significantly higher levels of IL-13 compared with those from pregnant women with the CC or CT genotype [21].

To our knowledge, the current study is the first to find a significant additive interaction between *IL13 *SNP rs1800925 and smoking that affects the risk of rhinoconjunctivitis, indicating a biological interaction, although the multiplicative interaction was not significant. Two risk factors are considered to engage in a biological interaction if they are involved in the same sufficient cause [22]. When the risk factors are independent and do not interact in a biological way, they are considered to have an additive effect [22]. The degree of a biological interaction between risk factors is measured as the difference between the observed effect and the expected additive effect of the interacting risk factors [22]. A previously cited study of UK adults observed a significant interaction between SNP rs1800925 and smoking with respect to allergy, but not with respect to asthma or hay fever: the relationship between SNP rs1800925 and allergy was stronger among smokers than among nonsmokers [5]. In the other 5 previously cited studies that have investigated *IL13 *SNPs and allergic rhinitis, gene-environment interactions were not examined [6-10]. In a study in Germany, children with the TT genotype of SNP rs1800925 or the AA genotype of SNP rs20541 who were exposed to postnatal maternal smoking had higher total serum IgE levels than similar children who were not exposed to maternal smoking, with statistically significant interactions between the *IL13 *variants and maternal smoking [23]. These results are in partial agreement with our findings. In contrast, a study of UK children revealed a significant interaction between SNP rs20541 and perinatal smoking exposure on the risk of early-onset persistent wheeze: the risk was most evident in children with the major GG genotype who were exposed to perinatal smoking [24].

The methodological strengths of this study include the homogeneity of study subjects in that they were all pregnant women, and the consideration of several confounders.

Several limitations also warrant mention. First, the participation rate cannot be calculated because the exact number of eligible pregnant women who were provided with the abovementioned KOMCHS documents is not available. In addition, we were not able to assess differences between participants and non-participants, because information on personal characteristics such as age, socioeconomic status, and a history of allergic disorders among the non-participants was not available. Our subjects were probably not representative of Japanese women in the general population: for example, the distribution of educational status among participants differed considerably from that among the general population. According to the 2000 population census of Japan, the proportions of women aged 30 to 34 years in Fukuoka Prefecture with years of education of < 13, 13 - 14, ≥ 15, and unknown were 52.0%, 31.5%, 11.8%, and 4.8%, respectively [25]. The corresponding figures for the current study in the control group were 21.9%, 33.0%, 45.1%, and 0.0%, respectively. Therefore, the present population might have had greater awareness concerning health-related matters compared to the general population. Although selection bias occurred, the distribution of the 2 SNPs under study in the control group was in agreement with the Hardy-Weinberg equilibrium.

Second, the definition of rhinoconjunctivitis was based on the questions in the ISAAC questionnaire. Validation tests of such questions have not been performed for Japanese young adults. Data on serum-specific IgE levels were not available. It is assumed that some proportion of cases may be misclassified, which could cause bias toward the null. However, the results of the sensitivity analysis confined to cases with doctor-diagnosed allergic rhinitis (n = 293) were similar to those in the overall analysis.

Third, the current study size, especially the number of cases, was rather small for a valid genetic association study. Although a significant association between SNP rs20541 and rhinoconjunctivitis and a significant additive interaction between SNP rs1800925 and smoking were identified, the insignificant additive interaction between SNP rs20541 and smoking might be attributable to insufficient statistical power in the current study.

Fourth, correction for multiple testing was not performed in this study. As this is a hypothesis testing study and part of the current findings is a replication of previously published results, we think that correction for multiple testing would cause us to underestimate our results. Despite this background, it should be noted that our findings might reflect a chance phenomenon, and that future studies will be needed to replicate the current new findings.

## Conclusions

The present case-control study of Japanese women showed that the AA genotype of *IL13 *SNP rs20541 and the haplotype comprising the rs1800925 C allele and the rs20541 A allele were significantly positively associated with the risk of rhinoconjunctivitis. Also, a new pattern of biological interaction that affects the risk of rhinoconjunctivitis is described between SNP rs1800925 and smoking. The current results, however, may not be generalizable to other populations. Given the strong epidemiological evidence supporting an environmental effect of smoking and our statistical suggestion of an interaction, further investigation is warranted to establish a biological mechanism of interaction.

## Abbreviations

CI: Confidence interval; ISAAC: International Study of Asthma and Allergies in Childhood; KOMCHS: Kyushu Okinawa Maternal and Child Health Study; OR: Odds ratio; SNP: single nucleotide polymorphism.

## Competing interests

The authors declare that they have no competing interests.

## Authors' contributions

YM, KT, and MA contributed to the study concept and design and the acquisition of data. YM was responsible for the analysis and interpretation of data and the drafting of the manuscript. All authors participated in critically revising the manuscript and approved the final version of the manuscript.

## Pre-publication history

The pre-publication history for this paper can be accessed here:

http://www.biomedcentral.com/1471-2350/12/143/prepub
